# ALLPATHS 2: small genomes assembled accurately and with high continuity from short paired reads

**DOI:** 10.1186/gb-2009-10-10-r103

**Published:** 2009-10-01

**Authors:** Iain MacCallum, Dariusz Przybylski, Sante Gnerre, Joshua Burton, Ilya Shlyakhter, Andreas Gnirke, Joel Malek, Kevin McKernan, Swati Ranade, Terrance P Shea, Louise Williams, Sarah Young, Chad Nusbaum, David B Jaffe

**Affiliations:** 1 Broad Institute of MIT and Harvard, Charles Street, Cambridge, MA 02141, USA; 2 Life Technologies, Cummings Center, Beverly, MA 01915, USA; 3 Current address: Department of Medical Genetics, Weill Cornell Medical College in Qatar, 24144 Al-Rayaan St., Doha, Qatar; 4 Current address: Pacific Biosciences, Adams Drive, Menlo Park, CA 94025, USA

## Abstract

We demonstrate that genome sequences approaching finished quality can be generated from short paired reads. Using 36 base (fragment) and 26 base (jumping) reads from five microbial genomes of varied GC composition and sizes up to 40 Mb, ALLPATHS2 generated assemblies with long, accurate contigs and scaffolds. Velvet and EULER-SR were less accurate. For example, for *Escherichia coli*, the fraction of 10-kb stretches that were perfect was 99.8% (ALLPATHS2), 68.7% (Velvet), and 42.1% (EULER-SR).

## Abstract 2

Allpaths2, a method for accurately assembling small genomes with high continuity using short paired reads.

## Background

Recent advances in sequencing technology [1-5] have rapidly driven down the cost of DNA sequence data. However, with the reduction in cost come challenges to using the data. The new technologies deliver novel data types that vary from each other in format. In addition, they are significantly different from traditional Sanger chemistry sequencing data in both read length and error rate, making it challenging to adapt them to many applications, including *de novo *assembly.

We consider here the problem of assembling short, high-quality reads, with the goal of producing genome sequences that are as good as or better than the 'draft sequence' quality that is the current standard. This standard is illustrated by two representative data sets: 87 bacterial genomes were recently sequenced using Roche/454 chemistry, having average contig and scaffold N50 (length-weighted median) lengths of 84 kb and 1.0 Mb, respectively (Table S1 in Additional data file 1); 13 bacterial genomes were sequenced by Sanger chemistry and subsequently finished, for which the draft sequences (prior to finishing) are, on average, 98.4% complete and have a base accuracy of less than 1 error per 10^4 ^bases (Table S2 in Additional data file 1). These data sets represent an appropriate standard of quality to which the community is accustomed and for which short read assemblies should strive. Recent work has begun to explore the possibilities of short read assembly [6-14], but high-quality assembly from experimentally generated paired reads has not been demonstrated, even for small genomes.

In this work we used reads from the Illumina platform generated from two libraries, yielding linking information of different sizes (approximately 200 and 4,000 bp). The inclusion of the library from longer fragments greatly increased the potential contiguity of the assemblies.

We previously demonstrated that high-quality assemblies could be obtained from simulated short paired reads [10]. We now demonstrate that high-quality assemblies can be obtained from real data as well. Our test cases consist of five finished and homozygous microbial genomes. Importantly, finished genome sequences represent an extremely high-quality standard with which to compare our assemblies, allowing us to evaluate the quality of our results precisely and rigorously. We also ran the assembly programs Velvet [12] and EULER-SR [9,14] on the same data sets and provide a side-by-side comparison.

Supplemental material is included with the paper. The Illumina sequence data, reference sequences, assemblies, and software used in this paper are freely available at [15,16]. The Illumina sequence data are also available from the NCBI Short Read Archive, using project identifiers 40071, 40073, 40075, 40077, and 40079.

## Results

We sequenced the genomes of three bacteria (*Staphylococcus aureus*, *Escherichia coli*, and *Rhodobacter sphaeroides*) and two fungi (*Schizosaccharomyces pombe *and *Neurospora crassa*) using the Illumina platform (Tables 1, 2, 3, 4 and 5; Table S3 in Additional data file 1). In all cases finished reference sequences were available. However, we found what appeared to be biological differences between our isolates and the reference sequences. There were only 11 differences in total for the first two bacteria, but 374 for *R. sphaeroides*. To provide a control data set for precisely evaluating our bacterial assemblies, we corrected the bacterial reference sequences using data from Illumina, then carefully validated the corrections using data from 454 and Sanger chemistries (Additional data file 1). For the fungi, we did not attempt to reconcile base-level differences, and thus, as compared to our samples, the accuracy of the reference sequences is lower.

**Table 1:** ALLPATHS, Velvet and EULER assemblies of five microbial genomes: source data

	*S. aureus*	*E. coli*	*R. sphaeroides*	*S. pombe*	*N. crassa*
**Source data**					
Strain	USA300	K12 MG1655	2.4.1	972 h	74A
Reference sequence	Finished, curated	Finished, curated	Finished, curated	Finished	Finished
GC composition (%)	33	51	69	36	49
Genome size (kb)	2,873	4,639	4,603	12,554	39,226
Reference N50 (kb)	2,873	4,639	3,188	4,509	665
Sequence coverage (x)	89	139	370	148	123

For five genomes, statistics from ALLPATHS, Velvet, and EULER assemblies are shown. In all cases identical data were supplied to both programs. Contigs of size <1,000 bases were discarded. Strain: strain that was sequenced. GC composition: percent of GC base pairs in the finished reference sequence. Genome size: total number of bases in reference. Reference N50: N50 size of reference. Reference sequences: all were finished, and in addition the reference sequences for the three bacterial genomes were curated to ensure full concordance with the samples, as described in the main text. *S. aureus*, circular chromosome and two plasmids; *E. coli*, circular chromosome; *R. sphaeroides*, finished but differs at 374 base positions from our sample, two circular chromosomes, four circular plasmids, and one linear plasmid; *S. pombe*, three linear chromosomes; *N. crassa*, finished at the base level, 251 contigs from 7 chromosomes. Sequence coverage: total coverage by usable reads; for a precise definition see Table S3 in Additional data file 1.

**Table 2:** ALLPATHS, Velvet and EULER assemblies of five microbial genomes: contiguity

	ALLPATHS	Velvet	EULER
**Contig N50 (kb)**			
*S. aureus*	477	87	68
*E. coli*	337	62	19
*R. sphaeroides*	156	151	3
*S. pombe*	51	55	24
*N. crassa*	19	16	14
			
**Scaffold N50 (kb)**			
*S. aureus*	611	562	68
*E. coli*	2,680	298	19
*R. sphaeroides*	858	1,126	3
*S. pombe*	222	422	24
*N. crassa*	58	186	14

For five genomes, statistics from ALLPATHS, Velvet, and EULER assemblies are shown. Contiguity refers to contig and scaffold N50 values.

**Table 3:** ALLPATHS, Velvet and EULER assemblies of five microbial genomes: genome coverage

	ALLPATHS	Velvet	EULER
**Covered by contigs ≥1 kb**			
*S. aureus*	99.1%	97.0%	96.7%
*E. coli*	99.3%	97.7%	94.6%
*R. sphaeroides*	98.5%	94.3%	65.0%
*S. pombe*	95.9%	95.5%	93.6%
*N. crassa*	89.5%	88.7%	89.2%
			
**Covered by contigs ≥10 kb**			
*S. aureus*	98.8%	94.4%	93.0%
*E. coli*	99.0%	92.4%	72.7%
*R. sphaeroides*	96.0%	87.2%	6.2%
*S. pombe*	91.3%	91.3%	76.3%
*N. crassa*	71.0%	62.1%	61.5%
			
**Covered by contigs ≥100 kb**			
*S. aureus*	88.3%	36.5%	41.3%
*E. coli*	86.5%	25.5%	0.0%
*R. sphaeroides*	69.5%	58.4%	0.0%
*S. pombe*	18.3%	17.8%	1.7%
*N. crassa*	1.3%	0.3%	0.0%

For five genomes, statistics from ALLPATHS, Velvet, and EULER assemblies are shown. Genome coverage refers to the fraction of the reference genome covered by the assembly, computed as the fraction of 100-mers in the reference sequence present in the assembly. If a 100-mer was present multiple times in the reference sequence, we checked for up to that many copies in the assembly.

**Table 4:** ALLPATHS, Velvet and EULER assemblies of five microbial genomes: correctness (of chunks approximately 10 kb or less)

	ALLPATHS	Velvet	EULER
**Class I: error rate 0**			
*S. aureus*	99.3%	70.7%	51.7%
*E. coli*	99.8%	68.7%	42.1%
*R. sphaeroides*	99.7%	71.8%	18.9%
*S. pombe*	79.7%	66.2%	31.4%
*N. crassa*	78.6%	49.9%	19.1%
			
**Class II: error rate <0.1%**			
*S. aureus*	0.7%	13.7%	26.4%
*E. coli*	0.2%	18.0%	24.1%
*R. sphaeroides*	0.0%	19.3%	39.2%
*S. pombe*	18.6%	26.6%	32.6%
*N. crassa*	15.3%	24.3%	24.1%
			
**Class III: error rate <1%**			
*S. aureus*	0.0%	9.1%	13.7%
*E. coli*	0.0%	6.4%	26.8%
*R. sphaeroides*	0.0%	5.9%	37.0%
*S. pombe*	1.3%	3.7%	28.7%
*N. crassa*	3.2%	11.4%	32.3%
			
**Class IV: error rate <10%**			
*S. aureus*	0.0%	6.2%	5.9%
*E. coli*	0.0%	5.9%	5.3%
*R. sphaeroides*	0.2%	2.0%	3.2%
*S. pombe*	0.3%	2.6%	5.1%
*N. crassa*	1.3%	7.9%	12.8%
			
**Class V: error rate ≥10%**			
*S. aureus*	0.0%	0.4%	2.3%
*E. coli*	0.0%	1.0%	1.7%
*R. sphaeroides*	0.1%	1.0%	1.5%
*S. pombe*	0.2%	0.8%	2.1%
*N. crassa*	0.9%	5.7%	10.4%
			
**Class VI: error rate, no match**			
*S. aureus*	0.0%	0.0%	0.0%
*E. coli*	0.0%	0.0%	0.0%
*R. sphaeroides*	0.0%	0.1%	0.2%
*S. pombe*	0.0%	0.0%	0.0%
*N. crassa*	0.7%*	0.9%*	1.4%*

For five genomes, statistics from ALLPATHS, Velvet, and EULER assemblies are shown. Correctness: contigs were divided into approximately 10 kb chunks, leaving smaller contigs intact. Subject to the caveat that the reference sequences might have some errors (likely greater for the fungi), we assayed the absolute accuracy of each chunk by finding the minimum number of errors (substitution or indel bases) among all alignments of it to the reference sequence for the genome. The table shows the distribution of the bases in the chunks according to their accuracy. Chunks having no 100-mer match are separately classified (class VI). *For *N. crassa*, some AT-rich regions that are missing from the reference [23] appear as novel sequence in the assemblies.

**Table 5:** ALLPATHS, Velvet and EULER assemblies of five microbial genomes: base accuracy, misassemblies, and long-range validity

	ALLPATHS	Velvet	EULER
**Base accuracy**			
Quality, from class I to III chunks			
*S. aureus*	Q59	Q33	Q32
*E. coli*	Q67	Q34	Q30
*R. sphaeroides*	>Q60	Q35	Q29
*S. pombe*	Q42	Q37	Q29
*N. crassa*	Q39	Q32	Q28
			
**Misassemblies**			
% in class IV to V chunks			
*S. aureus*	0.0%	6.6%	8.2%
*E. coli*	0.0%	6.9%	7.0%
*R. sphaeroides*	0.3%	3.0%	4.7%
*S. pombe*	0.5%	3.4%	7.2%
*N. crassa*	2.2%	13.6%	23.2%
			
**Long-range validity**			
At 100-kb distance			
*S. aureus*	100.0%	45.6%	99.8%
*E. coli*	100.0%	86.4%	N/A
*R. sphaeroides*	100.0%	75.8%	N/A
*S. pombe*	99.8%	80.2%	100.0%
*N. crassa*	99.8%	13.6%	N/A

For five genomes, statistics from ALLPATHS, Velvet, and EULER assemblies are shown. Base accuracy: the inferred quality score for the bases in chunk classes I to III, obtained by dividing the number of errors in these chunks by the total number of bases, and then taking -10*log_10 _of this quantity. Misassemblies: total fraction of bases in chunks in classes IV to V. Long-range validity: we chose 10,000 pairs of 100-base sequences at random from the scaffolds, where the two sequences were separated by 100,000 bases, considering only cases where both sequences could be placed uniquely on the reference. We scored the placement as 'valid' if both sequences were placed on the same chromosome, in the same orientation, with separation 100,000 ± 25% bases. We report the fraction of valid placements. N/A: all scaffolds were of length <100 kb.

The data for the assemblies were of three types: paired 36-base reads [5] derived from approximately 200-bp fragments, paired 26-base reads derived via a 'jumping' construction from approximately 4,000-bp fragments, and for one genome, additional unpaired 36-base reads. ALLPATHS requires, at a minimum, data from two paired libraries (ideally one with short distance spacing and one long). For the jumping library reads, we used a method (Additional data file 1) that we adapted from a protocol developed for the SOLiD platform [3]. This method first forms circles from the long fragments, placing a stuffer sequence at the junction site, then digests out the junction fragments using the *Eco*P15I restriction enzyme, which has fixed sites in the stuffer. Compared with protocols that work by randomly shearing the circles, this method has the advantage of yielding reads of known length and the disadvantage that the usable read length is only 26 bases.

The data were assembled using version 2 of ALLPATHS [10], using default arguments for all assemblies. For version 2, we introduced modifications that were essential for accurate handling of real data (Materials and methods). Conceptually, most of the modifications address the fact that genome coverage and coverage quality are sensitive to sequence context. One example is to determine whether a K-mer in the reads should be trusted as correct. Whereas the previous ALLPATHS version simply counted its number of copies in the reads, the new version instead uses quality scores to make the determination.

**Figure 1:**
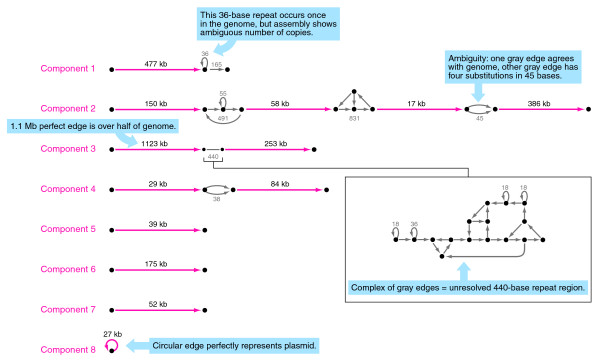
The ALLPATHS assembly of *S. aureus*. Each edge represents a contiguous and unambiguous sequence of bases and, for this assembly, each component is its own scaffold. Longer edges are in red, short edges in gray. The sizes of the gray edges and regions are in bases. Several key features are called out in blue boxes. Five short sequences totaling 9 kb are not shown. Images of the graphs for all five ALLPATHS assemblies of this paper are available at [16].

An ALLPATHS assembly is a graph, as shown in Figure 1 (*S. aureus*). A graph representation of an assembly consists of edges, representing contiguous and unambiguous sequences of bases, and vertices, representing junction points between edges, and thus branch points. Two edges exiting (or entering) a vertex define an ambiguity, which allows alternative reconstructions of an assembly region. Ambiguities typically arise from unresolved repeat sequences or heterozygous single nucleotide polymorphisms. Ideally, at least one of the reconstructions is correct.

The assembly graph may be divided into connected components, between which there are no edges. Using paired reads we may form scaffolds, which are linked sequences of one or more such components, separated by gaps. As part of the output of ALLPATHS we convert these graph scaffolds into traditional, linear scaffolds, which are presented via a fasta file with Ns for gaps. This standard output makes the data compatible with existing analytical tools. In these linear scaffolds, ambiguities (unresolved regions of the assembly graph) are replaced by gaps, entailing some loss of information. For example, the 36-bp repeat of Figure 1 is represented by a gap in the scaffold file, even though we know exactly what sequence is present in the gap, just not the exact number of copies. In contrast, for a true gap we have no knowledge of the missing sequence. Moreover, by treating all ambiguities as gaps we under-represent the true contiguity of the assembly.

Tables 2, 3, 4 and 5 quantify key properties of each assembly, including contiguity - how connected it is - and completeness - how much of the genome it covers. We also assessed the quality of the assemblies in several different ways: correctness, base accuracy, misassembly rate, and long range validity.

### Correctness

We devised an objective method to evaluate correctness of assemblies as follows. First, we broke each contig into approximately 10-kb chunks. (More precisely, each contig of size n > 10 kb was broken into ⌊ n/10^4 ^⌋ equal-sized chunks, ± 1 base. Contigs of size ≤10 kb were treated as separate chunks.) We then found the 'best' alignment of each chunk to the reference sequence, meaning the alignment that had the smallest total number of substitution and indel bases. We only considered alignments that subsumed a 100 base perfect match to the reference. (The special case where there was no alignment is described below.) From the best alignment we inferred the error rate of the chunk, understanding that these errors could either be errors in the assembly or in the reference sequence. Then we divided the chunks into six classes by their error rate. Class I contains the perfect chunks, class II chunks have error rates up to 0.1%, class III chunks have error rates up to 1%, class IV chunks have error rates up to 10%, and class V chunks have error rates of at least 10%. Class VI consists of chunks that appear not to match the reference at all, presumably due to either contamination of the sample or omissions from the reference, although very short and inaccurate chunks could also be in this category. We report the fraction of assembly bases in each class.

### Base accuracy

We assayed the 'base accuracy' of the assemblies by considering the error rate for bases in chunk classes I through III (error rate <1%). This translates to a PHRED-like quality score [17].

### Misassembly rate

The term 'misassembly' is typically used to refer to a relatively large defect, such as a rearrangement, but can also refer to a significant insertion, deletion or inversion. One way to precisely encapsulate this notion of large defect would be to declare a chunk misassembled if it has a sufficiently high error rate. We took this approach, declaring that a chunk was misassembled if it had an error rate of 1% or higher (that is, it was in class IV or V), reasoning that, given the depth of coverage and high accuracy of the sequencing technology, such a high error rate would most likely be the result of one or more large defects, rather than of isolated sequencing errors. We define the misassembly rate to be the fraction of bases in misassembled chunks. We note that all errors in chunks were accounted for either via base accuracy or misassembly; we took the division point to be at a 1% error rate.

### Long range validity

We assayed the long-range validity of the assemblies by computing the probability that sequences at distance 100 kb in scaffolds are, in fact, at about that distance in the genome (Table 5).

The ALLPATHS bacterial assemblies are highly contiguous, with N50 contig sizes ranging from 156 to 477 kb, and N50 scaffold sizes from 611 to 2,680 kb. The assemblies are nearly complete: coverage ranges from 98.5% to 99.3%. By all measures, the assemblies are highly accurate. The fraction of chunks (approximately 10 kb) that are perfect ranges from 99.3% to 99.8%. The inferred base accuracy is approximately Q60, that is, about one error in 10^6 ^bases. The long-range validity of the assemblies is perfect.

ALLPATHS is designed to present alternatives (graph branches) in cases where the exact sequence of the assembly cannot be determined. For example, as shown in Figure 1, component 2 of the assembly of *S. aureus *has two parallel edges representing a 45-base region, one of which matches the reference sequence perfectly, and the other of which has four substitutions. This ambiguity is distinguished from an error, where the assembly presents only the wrong sequence. The corrected reference sequences for the bacterial assemblies are nearly perfect, so we were able to generate a complete catalog of all errors in the ALLPATHS bacterial assemblies. There are only eight in a total of 12.1 Mb, as follows. The *S. aureus *assembly has exactly four errors: two single base mismatches separated by 11 bases, and two others separated by 23 bases. These events occur in repeat sequences of length >500 bp and >99% identity. The *E. coli *assembly has exactly one error: a single base mismatch. This event occurs in a perfectly repeated sequence of length 80 bp. The *R. sphaeroides *assembly has three errors. First, a 234 base deletion from the assembly, adjacent to a repeat. Second, a 10-kb component contains a 6.4-kb edge that matches a plasmid perfectly, but also a 3.4-kb edge that is misassembled and consists of repeated sequence. Third, a 160-kb component joins similar sequence between two plasmids, although all edges in the component match the reference sequence perfectly. This defect does not appear at all in the linearized scaffold.

For the ALLPATHS fungal genome assemblies, contiguity and completeness were lower than for the bacterial genomes. Thus, the N50 contig size for *S. pombe *was 51 kb, and for *N. crassa*, 19 kb. A lower fraction of the genome was represented: genome coverage was 95.9% for *S. pombe *and 89.5% for *N. crassa*. These assemblies were accurate, although not as accurate as the bacterial assemblies. Base quality was computed to be about Q40, although this is a floor estimate, since errors in the reference sequences or biological differences would have been reported as assembly errors. Long-range validity is very good: the odds of being correctly connected at distance 100 kb in a scaffold are about 99.8%.

Several factors can limit both the contiguity of assemblies and the fraction of the genome represented, including repeat sequence, extremes of GC composition, and non-equimolar sequences such as plasmids. We consider how these factors applied to two of the genomes: *R. sphaeroides *and *N. crassa*.

### *R. sphaeroides*

There was 98.5% of the genome present in the assembly; the missing regions consisted mostly of repetitive sequences. In our experience with the Illumina sequencing method employed here, representation decreases significantly for sequences of higher GC composition (Figure S1 in Additional data file 1), such as this genome (69% GC). We therefore sequenced very deeply to compensate for reduced coverage in GC-rich parts. The genome contains plasmids with two additional characteristics that challenged assembly: they are present at higher molarity than the chromosomes and contain long near-perfect repeat sequences.

### *N. crassa*

There was 89.5% of the genome present in the assembly. The 10.5% of the genome missing from the assembly is enriched in repetitive sequences and regions of very low GC composition.

In summary, for bacteria, the ALLPATHS assemblies were markedly complete, contiguous and accurate. The observed base accuracy of less than one error per million rivaled that of finished sequence [18,19]. Indeed, there were only five edges with any errors in the three bacterial assemblies. These assemblies were better than the accepted community draft standard. For the fungi, and especially *N. crassa*, the assemblies were accurate, but at a lower level of completeness and contiguity.

To understand how the ALLPATHS assemblies would compare to assemblies produced by existing software, we also assembled the identical datasets with Velvet [12] and EULER-SR [9,14], using standardized arguments for each assembler applied to all five genomes. In each case, we initially tested a range of arguments, with the goal of finding a single choice for settings that would optimize assembly quality. We note, however, that some choices optimized continuity at the expense of accuracy whereas other choices did the reverse. For Velvet and EULER-SR, we arrived at a single formula for each that was used in all assemblies presented here (Additional data file 1).

We first compare the results of ALLPATHS and Velvet (see Tables 2, 3, 4 and 5 for details). For *S. aureus *and *E. coli*, the ALLPATHS contigs were about five times longer than the Velvet contigs, whereas for the other three species, contig lengths were comparable. Scaffolds were longer for ALLPATHS for two species, and longer for Velvet for the other three; however, the ALLPATHS scaffolds were far more accurate. The odds of being correctly connected at distance 100 kb in an ALLPATHS scaffold were 100%, 100%, 100%, 99.8%, or 99.8%, depending on the species, whereas the same odds for Velvet were 45.6%, 86.4%, 75.8%, 80.2%, or 13.6%. In all five cases, the ALLPATHS assemblies were somewhat more complete. The base accuracy of the ALLPATHS assemblies was higher than for Velvet. For the bacterial genomes, where the reference sequences were essentially base perfect, thus enabling highly accurate measurement, we found that the ALLPATHS base quality was approximately Q60, whereas the Velvet base quality was approximately Q34. The reported base accuracies of the fungal assemblies were also higher for ALLPATHS. Finally, misassemblies were also much less frequent in the ALLPATHS assemblies: the misassembly rates for the ALLPATHS bacterial assemblies were 0%, 0%, and 0.3%, whereas those for the Velvet bacterial assemblies were 6.6%, 6.9%, and 3.0%. The ALLPATHS fungal assemblies also had about six-fold fewer misassemblies.

We also report results for EULER-SR assemblies (see Tables 2, 3, 4 and 5 for details). We note that EULER-SR scaffolding was minimal, yielding scaffolds of about the same size as contigs. In all cases EULER-SR contigs were shorter than either ALLPATHS or Velvet contigs. The EULER-SR assemblies were also substantially less accurate, in terms of both base accuracy and misassembly rate, than those produced by ALLPATHS and Velvet.

Finally, we carried out a series of 50 assemblies with the goal of understanding whether the results of Velvet or EULER-SR might be improved by using only the reads from approximately 200-bp fragments, thus excluding the shorter 26 base reads from long ('jumped') fragments, that might hinder the performance of the algorithms. For each of the two programs we tried two versions of the code, as well as multiple values of K: for Velvet we tried K = 25, 28, and 31, whereas for EULER-SR we used K = 25 and 28, the maximum allowed value. However, the EULER-SR assemblies with K = 28 terminated prematurely so we were unable to include the results.

Table S5 in Additional data file 2 displays the results of these auxiliary 'jump-free' assemblies. In brief, we note the following. Not surprisingly, scaffolds are much shorter for the auxiliary assemblies. For example, for *S. aureus*, the Velvet scaffolds are six-fold (or more) shorter than those in the assembly that uses all of the data. In some other ways, some of the assemblies from less data were better. For example, the auxiliary assembly of *R. sphaeroides *obtained from the older version of Velvet using K = 28 yielded contigs whose N50 size was 19% less than for the assembly of all the data, but which covered 2% more of the genome and were more accurate (0.5% misassembled versus 3%).

## Discussion

Here we demonstrate that high-quality assemblies of small genomes can be obtained from short reads, provided they are paired. At least for bacterial genomes, the ALLPATHS 2 assembly quality clearly exceeds that of draft assemblies from Sanger method data. Indeed, the base accuracy of 10^-6 ^greatly exceeds that of the finished sequence standard of 10^-4 ^originally set for the human genome (which has to come to represent a standard for genome finishing), and the standard of 10^-5 ^that was achieved [19]. Sequencing costs are already low enough that the same laboratory and computational methods might be applied to hundreds or thousands of such genomes. Given the current competitive environment among sequencing technologies, we expect costs to fall substantially further.

Difficult regions, such as recent duplications, will continue to pose a challenge, as they did with Sanger method sequencing. By creating assembly graphs, rather than linear assemblies (consisting of contigs and scaffolds), we can, in principle, capture these regions without fully disambiguating them [10,20]. Resolving these ambiguities will require improvements to the molecular biology, for example, very long reads.

In evaluating our results, we compared ALLPATHS to Velvet and EULER-SR, two other short read assemblers, on five data sets, and observed that the ALLPATHS assemblies are substantially more accurate. We note that all three programs were invoked with standardized settings for all genomes. Had we tuned the settings specifically for each genome using our knowledge of the reference sequences, performance would likely have been better in many cases, but the results less generalizable.

We now consider further improvements and new challenges for short read assembly. While we have demonstrated that nearly perfect bacterial assemblies can be generated from short reads, typically approximately 1% of the genome is missed. The fungal genomes, which are both more complex and five- to ten-fold larger, push the limits of short read assembly. Beyond this, we would like to assemble genomes even larger and more complex, such as those of mammals. Ingredients for success would include the following.

### Longer reads

Read lengths from Illumina's technology have increased to 100 bases in the past year, and longer reads are likely soon (data not shown).

### Better representation

By avoiding cloning bias, the new sequencing technologies can access regions that were previously recalcitrant, but they introduce new biases, for example by under-representing GC-rich regions. Early laboratory developments in this area are promising [21,22].

### Efficient library construction

Libraries with large numbers of distinct read pairs are required for assembly of large genomes. The key is to control process losses so that, from available amounts of starting material, sufficiently large libraries are produced. The importance of this increases proportionally with genome size as more read pairs are needed to cover the genome. We note that assembly algorithms must account for developments in laboratory methods. For example, a recent method [5] based on blunt end ligation rather than restriction generates jumping construct libraries of sufficient complexity for large genomes and not having a hard size limit on end reads. However, this method yields many reads containing the ligation point, thus posing a substantial algorithmic challenge.

### Scalable computation

Methods must be adapted so that the computational requirements for large genomes are feasible.

All of these ingredients could be at hand within a year, enabling genome assembly to fully cross the threshold from the Sanger method era to the 'short read' era, with comparable quality assemblies produced at approximately 100-fold lower cost.

## Materials and methods

ALLPATHS was first tested on simulated data [10]. Below we describe modifications that were needed for it to work well on real data.

### Removal of sequencing artifacts

We now discard all read pairs where both reads of the pair consist of 90% or more A bases. On the Illumina platform, such pairs are nearly always artifacts of the sequencing process, and on some runs can be sufficiently abundant as to cause problems for the assembly algorithms.

### Trusted K-mer identification

We identify putatively correct (or 'trusted') K-mers in the reads based on quality scores. This affects how we create unipaths and how we correct errors, as described below. For each K-mer that appears in the reads, we find all its instances in the reads, and examine the collection of read quality scores for a given base in the K-mer. That base is called trusted if there are enough good scores (default: 4 scores of at least 25). The entire K-mer is called trusted if each of its bases is trusted.

### Rehabilitation of K-mers between short fragment pairs

If for such a pair there is no path of trusted K-mers from one end to the other, but there is a path that uses some untrusted K-mers, we rehabilitate those K-mers, changing their status to trusted.

### Unipath creation

We find the (K+1)-mers in the reads whose first and last K-mer are trusted. The unipaths are mathematically defined by the trusted K-mers together with these (K+1)-mers, which define adjacencies between the trusted K-mers.

### Error correction

This has been modified to use the new definition of trusted K-mers.

### Unipath graph shaving

After the initial unipath creation, there are many cases in which read errors result in short terminal branches within the graph. Those shorter than 20 K-mers are now removed, provided that there is a longer alternative branch.

### Unipath recovery

This code identifies unipaths that are not represented in the assembly, extends them unambiguously where possible, and then adds them to the assembly. Typically this finds small regions that have relatively high copy number.

### ALLPATHS computational requirements

The five genomes were assembled on a 16-processor Dell server having 128 GB of memory. Some of the code is parallelized. The wall-clock times for the assemblies were: *S. aureus*, 1.7 hours; *E. coli*, 8.2 hours; *R. sphaeroides*, 10.2 hours; *S. pombe*, 80.5 hours; *N. crassa*, 86.6 hours.

## Abbreviations

N50: length-weighted median.

## Authors' contributions

AG, JM, KM, SR and LW developed methodology for *Eco*P15I ditag jumping libraries and constructed the libraries that were used. SY and TPS curated reference sequences and carried out assembly experiments using Velvet and EULER. SG and IM led ALLPATHS computational R&D. JB, DP and IS designed and implemented algorithms. DJ participated in R&D and wrote the manuscript. CN edited the manuscript and coordinated activities between groups. All authors read and approved the final manuscript.

## Additional data files

The following additional data are available with the online version of this paper: Tables S1 to S4, Figure S1, and several supplemental explanations (Additional data file 1); Table S5 (Additional data file 2).

## Supplementary Material

Additional data file 1 Tables S1 to S4, Figure S1, and several supplemental explanations. Click here for file

Additional data file 2 Table S5. Click here for file
