# Supplementary material for: ALLPATHS 2: small genomes assembled accurately and with high continuity from short paired reads
Source: Genome Biol. 2009 Oct 1;10(10):R103. doi: 10.1186/gb-2009-10-10-r103 (PMC2784318; doi:10.1186/gb-2009-10-10-r103)
Supplement: Additional data file 1 — Tables S1 to S4, Figure S1, and several supplemental explanations. [file gb-2009-10-10-r103-S1.PDF]

SUPPLEMENTAL

Table S1. Assembly statistics for 87 bacterial genomes recently sequenced by 454 technology

Organism	Sequence Coverage (X)	Assembly Size (Mb)	Fraction GC	Number of Scaffolds	Scaffold N50 (kb)	Number of Contigs	Contig N50 (kb)	Percent Q40*
Bacteroides fragilis 3_1_12	23	5.53	0.44	32	749	103	144	99.14
Bacteroides sp. 2_1_7	33	5.18	0.45	37	814	105	132	99.12
Bacteroides sp. 3_2_5	32	5.16	0.43	48	656	115	169	99.43
Bacteroides sp. 4_3_47FAA	22	5.45	0.43	88	176	212	79	97.70
Bacteroides sp. 9_1_42FAA	22	5.58	0.42	49	389	115	149	99.04
Bacteroides sp. D1	30	5.98	0.42	57	398	208	57	98.39
Bacteroides sp. D4	25	5.53	0.42	42	392	111	121	99.37
Bifidobacterium bifidum NCIMB 41171	33	2.20	0.63	9	1,500	33	106	99.21
Bifidobacterium longum CCUG 52486	34	2.48	0.60	22	476	55	171	98.82
Brucella abortus bv. 2 str. 86/8/59	42	3.29	0.57	21	1,920	130	49	98.79
Brucella abortus bv. 3 str. Tulya	43	3.28	0.57	13	780	60	122	99.28
Brucella abortus bv. 4 str. 292	32	3.27	0.57	12	778	47	180	99.43
Brucella abortus bv. 6 str. 870	41	3.27	0.57	13	777	55	123	99.42
Brucella abortus bv. 9 str. C68	41	3.27	0.57	13	777	50	135	99.37
Brucella ceti B1/94	35	3.34	0.57	14	1,910	102	54	98.17
Brucella ceti M13/05/1	32	3.34	0.57	22	910	118	47	98.33
Brucella ceti M490/95/1	38	3.35	0.57	17	1,210	142	39	97.87
Brucella ceti M644/93/1	38	3.33	0.57	17	909	104	68	98.45
Brucella melitensis bv. 1 str. Rev.1	32	3.31	0.57	26	1,170	93	94	98.89
Brucella melitensis bv. 3 str. Ether	41	3.31	0.57	13	1,180	105	61	99.12
Brucella neotomae 5K33	36	3.33	0.57	11	1,920	68	94	99.07
Brucella pinnipedialis B2/94	39	3.40	0.57	19	1,930	94	92	98.46
Brucella pinnipedialis M163/99/10	29	3.41	0.57	89	589	418	13	95.42
Brucella pinnipedialis M292/94/1	36	3.37	0.57	15	1,900	80	91	98.51
Brucella sp. 83/13	29	3.15	0.57	20	818	77	102	98.70
Brucella sp. F5/99	28	3.34	0.57	18	783	85	92	98.60
Brucella suis bv. 3 str. 686	25	3.30	0.57	23	782	129	48	99.01
Brucella suis bv. 5 str. 513	30	3.32	0.57	19	1,920	113	57	98.87
Citrobacter sp. 30_2	27	5.13	0.52	18	2,590	61	205	99.47
Clostridiales bacterium 1_7_47_FAA	29	6.55	0.50	108	1,260	172	162	99.22
Clostridium sp. 7_2_43FAA	30	3.84	0.28	29	3,240	132	67	98.30
Enterococcus casseliflavus EC10	30	3.38	0.43	16	729	51	166	99.11
Enterococcus faecalis AR01/DG	38	2.72	0.38	9	2,720	33	158	98.83
Enterococcus faecalis ATCC 4200	27	3.03	0.37	13	743	82	73	98.99
Enterococcus faecalis CH188	31	3.21	0.37	26	714	119	53	98.02
Enterococcus faecalis D6	36	2.90	0.37	10	1,710	44	173	99.27
Enterococcus faecalis DS5 (ATCC 14508)	32	2.99	0.37	39	437	100	70	98.78
Enterococcus faecalis E1Sol	42	2.80	0.38	12	1,490	66	83	99.17
Enterococcus faecalis Fly1	24	2.83	0.37	12	1,570	106	40	98.24
Enterococcus faecalis HIP11704	35	3.16	0.37	37	509	140	55	97.62

Enterococcus faecalis JH1	30	2.90	0.37	22	486	99	56	98.60
Enterococcus faecalis Merz96	21	2.99	0.38	19	1,520	99	51	98.41
Enterococcus faecalis T1	33	2.88	0.38	15	1,560	81	62	98.34
Enterococcus faecalis T11	29	2.68	0.38	12	1,460	46	113	99.27
Enterococcus faecalis T2	28	3.07	0.37	19	1,560	99	55	98.39
Enterococcus faecalis T3	39	2.72	0.38	9	1,470	38	142	99.68
Enterococcus faecalis X98	34	2.88	0.37	12	1,500	76	74	98.72
Enterococcus faecium 1,141,733	24	2.75	0.38	24	1,390	85	74	98.41
Enterococcus faecium 1,231,408	20	2.97	0.38	76	269	362	14	93.44
Enterococcus faecium 1,231,501	31	2.85	0.38	24	293	137	35	97.47
Enterococcus faecium 1,231,502	27	3.01	0.38	58	284	205	29	95.05
Enterococcus faecium Com12	27	2.71	0.38	19	485	67	82	98.87
Enterococcus faecium Com15	28	2.80	0.38	20	307	70	100	98.82
Enterococcus gallinarum EG2	24	3.16	0.41	14	477	49	202	98.98
Escherichia sp. 1_1_43	32	2.24	0.51	43	640	91	77	97.78
Escherichia sp. 4_1_40B	34	4.93	0.51	33	2,600	126	113	98.38
Francisella philomiragia ATCC25015	29	2.00	0.33	17	504	30	220	99.26
Fusobacterium gonidiaformans ATCC 25563	36	1.71	0.32	20	378	55	58	97.95
Fusobacterium mortiferum ATCC 9817	34	2.69	0.29	28	1,020	80	83	97.76
Fusobacterium sp. 2_1_31	24	2.53	0.28	35	805	202	23	94.79
Fusobacterium sp. 3_1_5R	37	1.93	0.32	28	966	99	44	95.65
Fusobacterium sp. 4_1_13	41	2.27	0.27	12	1,610	50	106	99.03
Fusobacterium sp. 7_1	29	2.51	0.27	18	1,450	95	45	98.26
Fusobacterium ulcerans ATCC 49185	34	3.50	0.30	47	525	123	64	97.97
Fusobacterium varium ATCC 27725	24	3.32	0.29	31	565	100	80	98.14
Helicobacter canadensis MIT 98-5491	49	1.63	0.34	23	890	126	23	98.24
Helicobacter cinaedi CCUG 18818	39	2.21	0.38	50	706	96	111	98.50
Helicobacter pullorum MIT 98-5489	60	1.95	0.34	44	277	131	43	98.20
Helicobacter winghamensis ATCC BAA-430	61	1.69	0.35	21	583	55	88	97.78
Lactobacillus jensenii 1153	23	1.76	0.35	11	248	57	113	96.67
Lactobacillus paracasei 8700:2	36	3.00	0.46	30	706	90	85	99.18
Neisseria gonorrhoeae 1291	38	2.11	0.53	41	627	174	21	96.21
Neisseria gonorrhoeae 35/02	42	2.13	0.53	39	1,410	155	23	95.71
Neisseria gonorrhoeae DGI18	39	2.11	0.53	41	532	152	23	96.23
Neisseria gonorrhoeae DGI2	39	2.17	0.53	37	672	132	37	96.18
Neisseria gonorrhoeae FA19	43	2.19	0.52	43	1,570	167	23	95.57
Neisseria gonorrhoeae FA6140	29	2.12	0.53	54	557	160	24	95.65
Neisseria gonorrhoeae MS11	40	2.20	0.52	44	1,580	195	20	95.34
Neisseria gonorrhoeae PID1	33	2.17	0.53	47	324	144	31	96.33
Neisseria gonorrhoeae PID18	25	2.17	0.53	48	625	174	23	95.73
Neisseria gonorrhoeae PID24-1	21	2.12	0.53	53	700	167	24	95.52
Neisseria gonorrhoeae PID332	42	2.19	0.52	46	660	167	24	95.62
Neisseria gonorrhoeae SK-92-679	30	2.12	0.53	45	620	195	20	95.11
Neisseria gonorrhoeae SK-93-1035	32	2.14	0.53	40	581	156	26	95.49
Oxalobacter formigenes HOxBLS	46	2.51	0.53	19	2,490	73	116	98.20
Oxalobacter formigenes OXCC13	49	2.44	0.50	9	2,420	27	220	99.18
Vibrio cholerae MO10	50	4.08	0.48	27	1,040	84	179	98.86

Averages	34	3.08	0.45	29.60	1,036	111	84	98.00

Table S1. Assembly statistics for 87 bacterial genomes recently sequenced by 454 technology. All assemblies included 20-fold or more sequence coverage. Genome data and statistics are available at [24]. *Percent Q40 = Percent of bases in the assembly that are labeled by the assembly software as being of Q40 or higher, which refers to an error rate of 1/10,000 or less.

Table S2. Bacterial assemblies from Sanger method reads

Organism	Genome size	Fraction GC	Assembled coverage (X)	Contig N50	Scaffold N50	% reference covered	Base errors	Consensus Q value
Acidithiobacillus ferrooxidans ATCC_53993	2,885,038	0.59	8.0	182,410	291,977	99.27%	123	43.7
Anaeromyxobacter sp. Fw109-5	5,277,990	0.74	7.7	135,441	5,267,785	99.59%	300	42.5
Dinoroseobacter shibae DFL_12*	4,417,868	0.66	7.9	132,717	3,786,660	97.85%	550	39.0
Fervidobacterium nodosum Rt17-B1	1,948,941	0.35	8.4	57,688	145,829	93.18%	211	39.7
Jannaschia sp. CCS1**	4,404,049	0.62	8.2	346,013	4,313,189	99.76%	452	39.9
Maricaulis maris MCS10	3,368,780	0.63	8.0	321,342	1,740,950	99.50%	269	41.0
Methanococcus maripaludis C5***	1,799,045	0.33	8.1	514,082	609,382	99.84%	208	39.4
Methanoculleus marisnigri JR1	2,478,101	0.62	8.0	266,013	2,445,998	99.67%	400	37.9
Parvibaculum lavamentivorans DS-1	3,914,745	0.62	8.1	418,517	1,299,215	99.83%	81	46.8
Psychromonas ingrahamii 37	4,559,598	0.40	8.0	225,323	4,537,440	98.38%	335	41.3
Pyrobaculum islandicum DSM_4184	1,826,402	0.50	8.1	247,966	984,127	99.52%	117	41.9
Thermoproteus neutrophilus V24Sta	1,769,823	0.60	8.2	380,545	1,188,075	98.87%	256	38.4
Thermotoga sp. RQ2	1,877,693	0.46	7.7	32,947	41,845	94.41%	207	39.6
Averages		0.55	8.0	250,846	2,050,190	98.44%	270	40.6

Table S2. The table shows statistics for several draft bacterial assemblies that were generated at the Broad Institute or the Whitehead Institute Center for Genome Research. All were subsequently finished, thus facilitating rigorous assessment. Genome size and fraction GC: computed from finished sequence. Assembled coverage: mean coverage of bases in the assembly by reads used in the assembly. Base errors: number of differences between the draft and finished sequences. Notes about genome sizes: *chromosome: 3789584, plasmids: 190506, 152970, 126304, 86208, 72296; **chromosome: 4317977, plasmid: 86072; ***chromosome: 1780761, plasmid 18285.

Table S3. Illumina sequence used in assemblies

Species	Flowcell	Lanes	Library type	Paired/ unpaired	Fragment size distribution (bp)	Read length	Bases (Mb)	PF bases (Mb)	PF Q20 bases (Mb)	Aligned PF Q20 bases (Mb)	Sequence coverage (x)
S. aureus	13229	1	fragment	paired	223 ± 11%	35	387	228	142	138	48.0
	201FK	5	jumping	paired	3848 ± 8%	26	202	144	125	121	42.1
E. coli	300AW	5-6	fragment	paired	210 ± 10%	35	1053	629	451	437	94.2
	201FK	1-2	jumping	paired	3776 ± 8%	26	283	220	205	202	43.5
R. sphaeroides	205E4	5-8	fragment	unpaired		36	1106	712	586	518	112.5
	205EF	5-8	fragment	unpaired		36	990	637	507	428	92.9
	13327	7-8	fragment	paired	185 ± 11%	35	505	334	177	160	34.7
	201G7	7	fragment	paired	205 ± 13%	35	374	248	184	178	38.6
	201FK	3,7-8	jumping	paired	3712 ± 8%	26	695	447	359	351	76.2
	13321	1	jumping	paired	3712 ± 8%	26	253	131	73	71	15.4
S. pombe	13329	7-8	fragment	paired	208 ± 11%	35	779	618	515	374	29.7
	202GC	5,7-8	fragment	paired	208 ± 11%	37	1240	971	626	451	35.9
	20B2U	2,5-8	jumping	paired	3655 ± 8%	26	2250	1612	1420	1029	81.9
N. crassa	13327	5	fragment	paired	210 ± 8%	35	197	152	94	85	2.1
	13350	3,5-6	fragment	paired	210 ± 8%	37	626	508	459	429	10.9
	202GC	1	fragment	paired	210 ± 8%	37	344	266	195	180	4.5
	202EA	1-3,5-8	fragment	paired	210 ± 8%	37	2163	1483	1272	1190	30.3
	201FN	1-3,5-8	fragment	paired	210 ± 8%	37	3951	2608	1494	1361	34.6
	13174	1-3,5-8	jumping	paired	3679 ± 10%	26	3218	2138	1846	1563	39.8

Table S3. Illumina sequence used in the assemblies. Flowcell: first five characters of the Illumina flowcell identifier. Lanes: lanes from the flowcell from which sequence was obtained. Library type: either fragment, meaning that reads were sequenced directly from the ends of a fragment, or jumping, meaning that a long fragment was circularized, the junction fragment isolated, and then the ends of it were sequenced. Paired/unpaired: whether one read (unpaired) or two (paired) were sequenced. Fragment size distribution: inferred fragment size distribution of library (paired reads only). Read length: the length of the reads in bases. Bases: total number of bases in the reads. PF bases: total number of bases in the purity-filtered (PF) reads, according to the Illumina pipeline's definition. PF Q20 bases: total number of PF bases that are scored Q20 or better by the Illumina pipeline. Aligned PF Q20 bases: total number of PF Q20 bases that are in reads having an alignment to the reference sequence for the genome with at most four differences. Sequence coverage: total coverage by usable bases, which we define to be aligned PF Q20 bases, divided by the reference genome size. This definition was taken as a heuristic proxy for coverage usable by the assembly algorithm.

Generation and validation of modified reference sequences

In order to have a high degree of precision in our analyses of the quality of ALLPATHS assemblies generated in this work, it was important to know the genome sequences as perfectly as possible. Because mutations do occur naturally at a very low rate, independent isolates derived from the same bacterial strain will almost inevitably differ at a few bases. In addition, finished genome sequences will contain a small number of errors, typically on the order of 1/100,000 bases [19], although the range is broad. Accordingly, we created a 'corrected reference' to represent the genome sequence of each of the exact bacterial samples that were sequenced and assembled for this work, and validated them using data from another sequencing technology (Roche/454) and followed up any unresolved bases with directed sequencing. The 'corrected references' were created by aligning deep Illumina sequences from our isolates of the bacterial genomes to the finished GenBank reference sequences for *E. coli*, *R. sphaeroides* and *S. aureus* (see Table S4 for accession numbers and a summary of all differences), and calling differences with our bacterial polymorphism caller VAAL [25]. Very high quality differences were then written into the GenBank reference sequences to create corrected reference sequences. Next, these corrected references were validated as follows. First, we aligned the corrected references to the GenBank references, and manually curated all 374 differences. Second, we created high quality deep sequence assemblies of the three genomes using an independent sequencing technology, Roche/454. Sequencing was performed by the 454's recommended methods on the FLX platform [26], and assembly was

performed with 454's Newbler assembler. The corrected references were aligned to the 454 assemblies, and all differences were manually curated. Third, all sequence differences from the GenBank/corrected reference comparison and the corrected reference/454 comparison were compared. The 337 GenBank/corrected reference differences that were corroborated by corrected reference/454 differences were considered to be validated as true differences between the isolates used for the finished references and the isolates sequenced in this work. For regions of the genomes that were not covered by assembled 454 data, unassembled 454 reads were aligned to the genome and differences called. This validated a further 8 base differences. This analysis accounted for all of the differences between the *E. coli* isolates, all but a single A/T base difference between the *S. aureus* isolates, and 335 of 363 differences between the *R. sphaeroides* isolates. Fourth, the remaining 28 differences between the *R. sphaeroides* isolates were resequenced directly by PCR amplification and double-ended Sanger chemistry sequencing. This was done in triplicate, using three primer pairs for each difference. With two exceptions, all *R. sphaeroides* base differences were validated. These exceptions included a region in chromosome 1 that we were unable to amplify by PCR that contained a single base difference and a region at the end of plasmid A that contained 4 base differences. Because the reference sequence for plasmid A is linear and the base differences occurred at the end of the reference sequence, we were unable to design primers flanking the region containing the differences.

Table S4. Validation of corrected reference sequences

Organism	Chromosome or plasmid	GenBank reference	Differences between corrected reference and GenBank	Confirmed by 454 Assembly	Confirmed by 454 Reads	Confirmed by PCR + Sanger	Corrected reference wrong	Unverified
R. sphaeroides	1	NC_007493.1	297	285	1	10	0	1*
	2	NC_007494.1	45	45	0	0	0	0
	plasmid A	NC_009007.1	6	0	2	0	0	4**
	plasmid B	NC_007488.1	12	1	0	11	0	0
	plasmid C	NC_007489.1	2	0	0	2	0	0
	plasmid D	NC_007490.1	1	1	0	0	0	0
	plasmid E	NC_009008.1	0	0	0	0	0	0
S. aureus	1	NC_010079.1	2	2	0	0	0	0
	pUSA300HOUMR	NC_010063.1	3	2	0	0	0	1***
E. coli	1	U00096.2	6	1	5	0	0	0

Table S4. Differences between GenBank reference sequences and modified versions matching our isolates were validated using alternate sequencing technologies, as described in the supplemental text. The table provides an accounting of this process. Notes: *PCR failed in this region, **Region is at the end of a linear plasmid so could not be amplified by standard PCR, ***Validation was not attempted for this position.

For *S. pombe* and *N. crassa*, we used the available reference sequences without any changes. These were GenBank AL672256.4 + AL672257.4 + AL672258.3 + X54421.1 and GenBank AABX02000000, respectively.

Construction of EcoP15I ditag jumping libraries

EcoP15I ditag jumping libraries were constructed following a modified version of the protocol originally developed for SOLiD mate-pair sequencing [27].

Genomic DNA (15 µg) in 125 µl TE0.1 buffer (10 mM Tris-HCl, pH 8, 0.1 mM EDTA) was mechanically sheared using the Digilab HydroShear device by 30 passages through an 0.0025-inch orifice at speed code 13

and incubated for 30 min. at 20˚C in a 200 µl End-It end-repair reaction (Epicentre). Samples were cleaned up on two QIAquick PCR purification spin columns and eluted in a total of 150 µl EB buffer. Next, EcoP15I recognition sites in the genomic DNA were methylated by incubation for 90 min. at 37˚C in a 200 µl volume with 750 units/ml EcoP15I (NEB) in 1x NEBuffer 3 containing 100 µg/ml acetylated BSA and 0.38 mM S-adenosyl methionine (NEB). Reactions were inactivated by heating at 65˚C for 20 min., and the reaction volume was increased to 300 µl by addition of 500 pmol EcoP15I adapters with non-self-complementary TGAG-3' overhangs (pre-annealed from 5'-[Phos]CTCAGCAG and 5'-[Phos]CTGCTGAGTGAG), ATP (1 mM final concentration) and 25 units T4 DNA ligase (Ambion). After incubation for 1 h at 20˚C, samples were cleaned up on two QIAquick PCR purification spin columns. Next, adapter-ligated fragments were run at 25 V overnight on a 1% agarose gel in 1xTAE. The SYBR green-stained DNA smear was visualized on a DarkReader (Clare Chemical) and the gel slice containing fragments in the size range from 3.5 to 4.5 kb excised and solubilized at room temperature with 3 volumes of QG buffer (Qiagen). Size-selected fragments were purified on QIAquick spin columns (Qiagen), eluted in 200 µl EB buffer and quantified by NanoDrop spectrophotometry.

Gel-purified ~4 kb fragments (typically ~2.5 µg, *i.e.* ~1 pmol) were circularized at a concentration of 0.65 ng/µl in the presence of 3 pmol biotinylated circularization adapter (pre-annealed from 5'-[Phos]CTAGTACA[Biotin-dT]CATGCCTCA and 5'-[Phos]GCATGATGTACTAGCTCA) with CTCA-3' overhangs that are complementary to the TGAG-3' overhangs on the EcoP15I-adapter-ligated genomic DNA fragments. At this concentration, the expected ratio of circularization of single ~4-kb fragments to concatenation events involving two different ~4 kb fragments is approximately 50:1 [28]. Ligations (typically ~4 ml) in 1x T4 DNA ligation buffer (NEB) containing 12.5 units/ml T4 DNA ligase (Ambion) were incubated overnight at 16˚C. To degrade linear DNA molecules, 10 units "plasmid-safe" ATP-Dependent recBCD nuclease (Epicentre) per µg of ~4 kb DNA fragments and fresh ATP (0.14 mM f.c.) were added and the reaction incubated for 40 min. at 37˚C. Nuclease-resistant (circular) DNA was purified on a Concentrator-100 spin column (Zymo Research), eluted in 150 µl TE0.1 buffer and quantified by NanoDrop spectrophotometry.

Purified circularized DNA (typically, 10-20% of the genomic DNA going into the circularization) was digested overnight at 37˚C in 240 µl 1x NEBuffer 3 supplemented with 100 µg/ml acetylated BSA, 0.1 mM Sinefungin (Sigma), 2 mM ATP and 500 units/ml EcoP15I (NEB). After addition of fresh ATP (20 µmol), Sinefungin (10 nmol) and EcoP15I (50 units) and an additional hour at 37˚C, the 250 µl digestion reaction was stopped by heating 20 min. at 65˚C and then placed on ice. End-It (Epicentre) 10x end-repair buffer, 10 mM ATP, 2.5 mM dNTPs (37 µl each) and end-repair enzyme mix (4 µl) were added. After 45 min. at 20˚C, end-repaired fragments were purified on a QIAquick MinElute column. To the 30 µl volume of the eluate we added 5 µl 10x NEBuffer 2, 10 µl 1 mM ATP, 2 µl H2O and 3 µl 5 units/µl exonuclease-deficient large Klenow fragment (NEB). After incubation for 30 min. at 37˚C, the enzyme was heat inactivated for 20 min. at 65˚C. Illumina paired-end adapters (typically 6 to 12 pmol, that is, ~60 molecules per molecule of ~4 kb circle present before the EcoP15I digest), 5 µl 10 mM ATP and 2 µl 400 units/ul T4 DNA ligase (NEB) were added. After 2 h at 20˚C, the ligation mix was diluted with 240 µl TE0.1 buffer.

To isolate EcoP15I ditags attached to the biotinylated circularization adapter, 30 µl MyOne Streptavidin C1 beads (Invitrogen) were washed twice with 200 µl TTNE buffer (0.1% Tween-20, 10 mM Tris-HCl, pH 8.0, 2 M NaCl, 1 mM EDTA), resuspended in 300 µl TNE (10 mM Tris-HCl, pH 8.0, 2 M NaCl, 1 mM EDTA) and added to the diluted ligation mix. After 15 min. with gentle agitation at 20˚C, the beads were pulled down and – after discarding the supernatant – resuspended in 400 µl TTNE and transferred to a fresh microcentrifuge tube.

The beads were collected, washed once with 400 µl TNE and twice with 100 µl 1x NEBuffer 2 and resuspended in 50 µl 1x NEBuffer 2.

Four trial PCR reactions, each containing 0.6 µl bead-immobilized EcoP15I ditag library and Illumina PE1.0 and PE2.0 PCR primers (1.5 pmol each) in 10 µl 1x Phusion High Fidelity master mix with HF buffer (NEB), were set up to determine the number of cycles necessary to generate enough PCR product for sequencing. The temperature profile was 30 s at 98°C followed by 12, 15, 18 or 21 cycles of 10 s at 98°C, 30 s at 65°C, 30 s at 72°C and a final 7-min. extension at 72°C. The remainder of the bead-immobilized library was amplified for 12-15 PCR cycles in a preparative 200 µl (4 x 50 µl) reaction with 125 pmol each of Illumina PE1.0 and PE2.0 PCR primers. The 211-bp PCR product was purified on a preparative 3% NuSieve 3:1 agarose gel (Lonza) followed by QIAquick gel extraction.

Canonical cleavage with EcoP15I generates a double-strand break with a two-base 5'-overhang 27 bases from the recognition site. By traditional Sanger sequencing we found shorter 26-base tags at about half the frequency as canonical 27-base tags. We therefore trimmed the tags conservatively after 26 bases.

Figure S1. GC vs coverage in sliding 100 bp windows

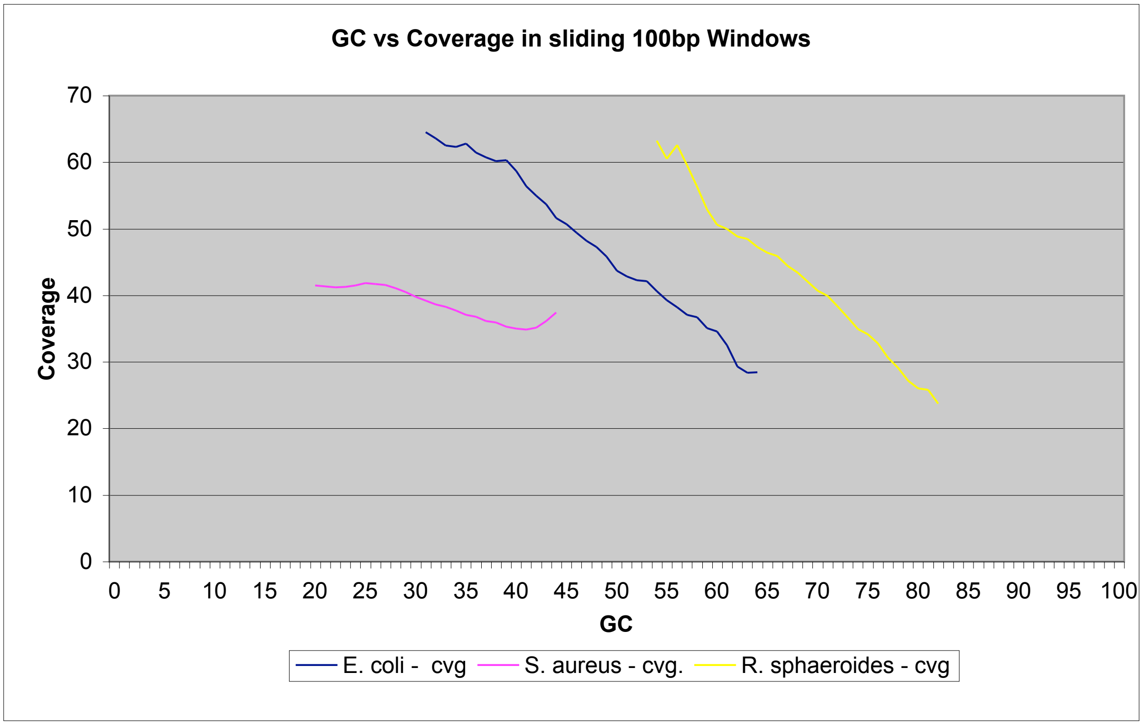

Figure S1. For the bacterial species assembled in this paper (*S. aureus*, *E. coli*, and *R. sphaeroides*), we show coverage as a function of GC composition. For each species, reads totaling roughly 50x coverage were taken from one lane (flowcell.lane = 13229.1, 300AW.5, 201G7.7, as in Table S3). For GC compositions 0%, 1%, ..., 100%, we scanned the genome of each species to find all 100 bp windows having that GC composition. For a given species and GC composition, if there were at least 20,000 such windows, we plotted a point showing the mean read coverage corresponding to that GC composition.

Invocation of Velvet

There were several user-supplied parameters that could be set. As we were unsure of the optimal value for these parameters, we experimented with several settings, with the goal of finding settings that would optimize assembly quality. We found that the exp_cov parameter was critical. Choosing a very low value produced highly accurate assemblies that were however less contiguous than the assemblies resulting from a higher value. Contigs had an N50 size that was two to three fold smaller. We chose an intermediate value for the parameter that yielded *relatively* high continuity and accuracy.

We used version 0.7.30. Reads from the jumping library were reverse complemented so that the pairs presented to Velvet would face inward. We first ran Velveth with hash_val = 25. Then we ran Velvetg without supplying any parameters. From its output, we obtained a value for the average coverage of contigs, considering only those contigs which were above the N50 contig size. Then we ran Velvetg again, this time assigning to the parameter exp_cov the value for average coverage obtained from the first run, and in addition making the following parameter choices: cov_cutoff = 5, ins_length = 200, ins_length2 = 4000, and min_contig_lgth = 100. This exact same procedure was followed for all five genomes.

We parsed the Velvet output file as follows. First, fasta records were understood to be scaffolds. Then, whenever a fasta record had a sequence of one or more Ns, we discarded the Ns and broke the fasta record in two. (We determined empirically that single Ns do in fact correspond to gaps, rather than ambiguous bases.)

Invocation of EULER-SR

We used version 1.1.1 of EULER-SR. (This is the same as EULER-USR.) We following the instructions in the file README.eulersr that is part of the EULER-SR distribution. As per these instructions, reads were quality trimmed using qualityTrimmer and further filtered using filterIlluminaReads. Each assembly used a vertex size of 25. Other parameters choices were the default.
