# Supplementary material for: ALLPATHS 2: small genomes assembled accurately and with high continuity from short paired reads
Source: Genome Biol. 2009 Oct 1;10(10):R103. doi: 10.1186/gb-2009-10-10-r103 (PMC2784318; doi:10.1186/gb-2009-10-10-r103)
Supplement: Additional data file 2 — Table S5. [file gb-2009-10-10-r103-S2.PDF]

Table S5. Assemblies using only reads from ~200 bp fragments

species	prog	version	K	contig size (N50)	scaff-old size (N50)	% cover -age by contigs ≥ 1kb	% cover -age by contigs ≥ 10kb	% cover -age by contigs ≥ 100kb	class I (%)	class II (%)	class III (%)	class IV (%)	class V (%)	class VI (%)	base accur -acy	mis-assembly rate (%)	long range validity (%)
S.aureus	Velvet	0.7.17	25	55173	87428	96.7	91.3	36.5	78.3	15.6	4.03	1.33	0.73	0	Q39	2.06	68.5
S.aureus	Velvet	0.7.47	25	55173	87428	96.8	91.4	37.7	78.8	15.6	3.09	1.36	1.09	0	Q39	2.45	77.5
S.aureus	Velvet	0.7.17	28	46014	73003	96.8	91.1	20.5	81	13.4	3.57	1.06	0.95	0	Q38	2.01	95.2
S.aureus	Velvet	0.7.47	28	46295	73003	96.8	91.1	20.5	82	13.4	3.57	1.06	0	0	Q38	1.06	95.7
S.aureus	Velvet	0.7.17	31	2647	47246	73.1	1.06	0	89.6	7.5	1.36	1.41	0.12	0	Q42	1.53	76.5
S.aureus	Velvet	0.7.47	31	2647	45754	73.1	1.06	0	89.6	7.5	1.36	1.41	0.12	0	Q42	1.53	86.3
S.aureus	EULER	1.1.1/2	25	43091	45764	96.3	90.1	17.9	57	28.8	10.5	3.15	0.5	0	Q34	3.65	100
E.coli	Velvet	0.7.17	25	59947	80436	96.8	91.7	30.4	73.5	23.3	2.27	0.51	0.48	0	Q40	0.99	85.9
E.coli	Velvet	0.7.47	25	63471	82713	96.8	91.9	33.1	74.5	23	1.74	0.51	0.26	0	Q41	0.77	100
E.coli	Velvet	0.7.17	28	46595	95387	96.8	89.7	15.8	81.6	15.1	1.97	1.31	0	0	Q42	1.31	91.5
E.coli	Velvet	0.7.47	28	47488	95387	96.8	89.8	15.8	81.4	15.3	1.99	1.31	0	0	Q41	1.31	90.7
E.coli	Velvet	0.7.17	31	5806	79536	87.5	20.5	0	76.5	19.5	3.86	0.19	0	0	Q38	0.19	91.3
E.coli	Velvet	0.7.47	31	5809	80877	87.5	20.5	0	77	19	3.72	0.1	0.14	0	Q38	0.24	100
E.coli	EULER	1.1.1/2	25	18258	32000	94.4	71.6	0	33.4	17.5	39.6	7.85	1.64	0	Q27	9.49	100
R.sphaeroides	Velvet	0.7.17	25	82330	120360	94	85.5	34.6	72.9	22.1	2.59	0.93	1.4	0.12	Q39	2.33	66.8
R.sphaeroides	Velvet	0.7.47	25	38859	42563	93.9	81	10.3	75.7	21.4	2.71	0	0	0.12	Q39	0.00	100
R.sphaeroides	Velvet	0.7.17	28	122500	143984	96.3	89.3	66.6	84	12.2	3.14	0	0.51	0.12	Q38	0.51	84
R.sphaeroides	Velvet	0.7.47	28	80210	106766	96.3	88.9	42.2	85.6	12.1	2.15	0	0.05	0.12	Q41	0.05	99.5
R.sphaeroides	Velvet	0.7.17	31	47708	67613	96.9	90.1	16.3	79.4	17.6	1.75	0.86	0.23	0.08	Q40	1.09	81.2
R.sphaeroides	Velvet	0.7.47	31	43892	63340	96.9	89.6	12.8	80.5	17.6	1.33	0.54	0	0.08	Q41	0.54	100
R.sphaeroides	EULER	1.1.1/2	25	16481	24861	48.5	35.3	0	2.89	41.4	47.7	7.2	0.57	0.22	Q27	7.77	N/A
S.pombe	Velvet	0.7.17	25	45254	122516	95	89	9.72	65.7	30	2.77	0.9	0.63	0.01	Q39	1.53	77.7
S.pombe	Velvet	0.7.47	25	45520	123706	95	89.2	11.9	64.8	31.9	2.73	0.34	0.25	0.02	Q38	0.59	99.3
S.pombe	Velvet	0.7.17	28	32479	167303	94.9	85	1.06	64	31	3.32	0.39	1.28	0.05	Q37	1.67	83.1
S.pombe	Velvet	0.7.47	28	31732	169668	94.9	84	1.06	64.9	32.1	2.23	0.19	0.53	0.05	Q38	0.72	99.1
S.pombe	Velvet	0.7.17	31	4786	125805	87.2	9.81	0	72.6	21.2	4.19	1.49	0.34	0.11	Q37	1.83	93.5
S.pombe	Velvet	0.7.47	31	4783	143652	87.2	9.81	0	72.5	21.7	4.45	1.04	0.23	0.11	Q37	1.27	97.5
S.pombe	EULER	1.1.1/2	25	18535	38784	93.2	72.8	0	21.5	22.7	46	7.63	2.15	0.04	Q27	9.78	100
N.crassa	Velvet	0.7.17	25	17564	54213	91.1	68.6	0	39.9	29.6	16.9	6.41	5.92	1.3	Q31	12.33	30.3
N.crassa	Velvet	0.7.47	25	18238	51481	91.4	70.3	0	40.2	34.8	20.6	1.82	1.01	1.55	Q31	2.83	91.8
N.crassa	Velvet	0.7.17	28	18488	56301	90.7	69.2	0.64	44.1	33.8	11.8	4.39	4.84	1.15	Q33	9.23	54
N.crassa	Velvet	0.7.47	28	18611	53550	91.1	70.4	0.35	45.3	38	13.1	1.62	0.75	1.32	Q33	2.37	90.4
N.crassa	Velvet	0.7.17	31	7633	50267	83.7	30.3	0	58.1	29.1	8.93	2.14	1.56	0.2	Q34	3.70	67.9
N.crassa	Velvet	0.7.47	31	7633	47990	83.8	30.3	0	58.6	30.2	9.34	1.32	0.32	0.22	Q34	1.64	86.1
N.crassa	EULER	1.1.1/2	25	13293	15482	89.4	59.9	0	21.5	25.4	36	10.1	5.82	1.26	Q28	15.92	100

Table S5. For five genomes, we assembled the same data as in Table 1, except that only the reads from ~200 bp fragments were used; reads from jumping libraries were thus excluded. The arrangement of this table mirrors Table 1, although it is transposed. We used both Velvet and EULER. We did not use ALLPATHS because it is not designed to work with data from only one library. For each of the two programs we used two versions of the code, for Velvet, 0.7.17 and 0.7.47, and for EULER, 1.1.1 and 1.1.2. The results from the two versions of EULER were identical so we present the results for two runs as a single line. For Velvet, we used three values of K: 25, 28, and 31. For EULER, we used 25 and 28 (the maximum allowed value), however the assemblies for K = 28 terminated prematurely, so we do not show the results.
